# Environment and Host Genetics Drive Infection Outcomes in a Butterfly Host, but Do Not Interact With Each Other

**DOI:** 10.1002/ece3.74075

**Published:** 2026-08-03

**Authors:** Ania A. Majewska, Grace Chlebove, Jacobus C. de Roode

**Affiliations:** ^1^ Southeastern Cooperative Wildlife Disease Study, Department of Population Health College of Veterinary Medicine, University of Georgia Athens Georgia USA; ^2^ Department of Physiology and Pharmacology College of Veterinary Medicine, University of Georgia Athens Georgia USA; ^3^ O. Wayne Rollins Research Center, Department of Biology Emory University Atlanta Georgia USA

**Keywords:** Asclepias, *Danaus*, diet, G × E, pathogen

## Abstract

Understanding how host genetics and the environment interact to impact host resistance to parasite infection is crucial for predicting and controlling infectious diseases. Here we examined how different host families and environmental conditions (as provided by larval food plant) impact the probability of infection, infection burden, host lifespan, and size in a butterfly host. We used the monarch butterfly, *Danaus plexippus* , and two species of milkweed, *Asclepias curassavica* and *A. incarnata* , to examine the outcomes of different host genotypes (31 full‐sib families) in response to inoculation with a common and naturally occurring protozoan parasite, *Ophryocystis elektroscirrha.* We tested for the significance of genotype and environment (G × E) interaction effects, as well as the main effects of genotype and environment. We found that plant species affected infection status, infection burden, and host lifespan, and that host genetics impacted lifespan and wing length. However, our results indicated no G × E effects on any infection outcomes, indicating that larval diet and genetic effects operate independently in this system.

## Graphical abstract

This study examined how host genetics and larval food plant environment influence parasite infection outcomes in monarch butterflies ( *Danaus plexippus* ) infected with the protozoan *Ophryocystis elektroscirrha*. We tested for genotype × environment interactions on infection status, infection burden, lifespan, and wing size. Milkweed species affected infection outcomes and lifespan; host genetics influenced lifespan and wing length, but no genotype × environment interactions were detected, suggesting these factors act independently.

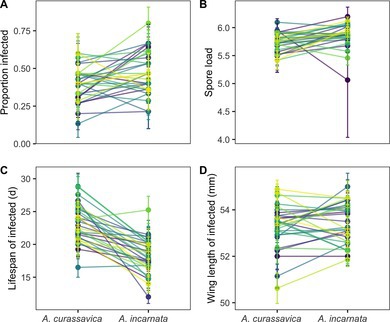

## 1 Introduction

The relative contributions of genetic effects and environmental variables and their interaction on susceptibility and resistance to pathogens remain central to understanding disease ecology and evolution (Lively et al. 2014; Read et al. 2015). Among individuals and populations, genetic differences in behavioral and physiological defenses against a pathogen affect exposure, likelihood of becoming infected, pathogen load, and the severity of pathogen‐induced damage (Howick and Lazzaro 2014; Oyesola et al. 2024). Yet, as with many other traits, the environment can mediate these outcomes (Sandland and Minchella 2003; Wolinska and King 2009; Sirovy et al. 2021). The environment imposes a suite of abiotic and biotic conditions that can modify the relative contributions of genetics to disease outcomes (Scholthof 2007; Hoffmann and Merila 1999; Charmantier and Garant 2005). For example, across populations of lowland leopard frogs ( *Lithobates yavapaiensis* ), temperature alone determines infection intensity with a fungal pathogen *Batrachochytrium dendrobatidis*, while both genetic diversity and temperature influence the probability of infection (Savage et al. 2015). Thus, the environment can amplify or diminish the expression of genetic effects on disease susceptibility and thereby shape host‐pathogen interactions.

Among environmental factors, diet is particularly important because it impacts host physiology and thereby can alter parasite growth, transmission, and virulence (Lange et al. 2014; Sarfraz et al. 2009; Bukovinszky et al. 2009; Miller and Cotter 2018). Across taxa, food quality, or the nutrient content in relative amounts of carbon, nitrogen, and phosphorus as well as micronutrients, can directly influence host defenses (Calder and Kulkarni 2017; Cotter and Al Shareefi 2022; Smith 2007). High‐quality food can enhance immune responses and benefit the host in clearing and controlling the pathogen infection (Wiehn and Korpimäki 1998; Klasing 1998; Childs et al. 2019). Wild birds, for instance, with greater dietary protein show higher tolerance to *Mycoplasma gallisepticum* infection (Sauer et al. 2025). On the other hand, a poor‐quality diet can impair immunity by disrupting immune cell development, leading to increased susceptibility and morbidity, and longer infectious periods (Cotter et al. 2019; Klasing 1998; Cotter et al. 2011). Thus, resistance and tolerance to pathogen infection are dependent upon the quality of the diet consumed by the host.

In herbivores, diet is shaped not only by nutritional content but also by secondary plant chemistry (Cotter and Al Shareefi 2022; Cory and Hoover 2006; Sanaei and de Roode 2025). Specifically, plant secondary compounds, intended to deter the herbivores from feeding on the plant tissues, can influence herbivore's immune function and physiology. For instance, when cabbage white ( *Pieris rapae* ) caterpillars feed on plants with higher glucosinolate concentrations, caterpillars show greater defense against a parasitoid through enhanced encapsulation response (Ghosh et al. 2023). Yet, toxins can act as stressors and mediate the immune system activation, directly suppressing immune function expression and pathogen replication (Smilanich et al. 2009; Tan et al. 2019; Ode and Ghosh 2025; Sternberg et al. 2012), or indirectly change disease outcomes by reducing host consumption and digestion and thereby decreasing host growth and survival (Chen 2008; Couture et al. 2016; Paul et al. 2021). The direct and indirect diet‐mediated effects on pathogen resistance and tolerance might be particularly important for specialist herbivores that have co‐evolved with a specific plant species and their chemistries. However, for specialist herbivores, how host genetic variation interacts with dietary variation in plant chemistry to influence disease outcomes remains poorly understood.

Here we use the monarch butterfly ( *Danaus plexippus* ), its larval food plants (*Asclepias* spp.), and specialist parasite *Ophryocystis elektroscirrha* (OE) as a well‐suited system for examining how host genetics and diet interact to determine infection outcomes. Monarchs are susceptible to infection with the protozoan parasite OE which begins at the larval stage when a larva ingests OE spores along with its food plant, milkweed (Mclaughlin and Myers 2007). OE infection in monarchs is characterized by negative impacts on flight performance, fecundity and mating success, and lifespan (Bradley and Altizer 2005; Lindsey and Altizer 2009; Altizer and Oberhauser 1999; de Roode et al. 2009). Previous work has shown that monarch full‐sib families vary in resistance to OE infection and immune responses (Lefèvre et al. 2012). Another source of variation comes from the larval food plant: monarch females oviposit and larvae exclusively feed on milkweed species (mostly in the genus *Asclepias*), which vary widely in the concentrations and diversity of secondary compounds, cardenolide toxins (Agrawal et al. 2012; Agrawal et al. 2015). While cardenolides are toxic to most animals, monarchs evolved to bypass the toxins and sequester them in their tissues as anti‐predator defense, although there is some negative impact on fitness with high concentrations (Agrawal 2005; Zalucki and Brower 1992; Brower et al. 1968; Petschenka et al. 2013; Tao et al. 2016). In addition to conferring anti‐predator protection, previous studies have shown that cardenolides also influence monarch infection outcomes: larvae that feed on plant species with high diversity and concentrations of cardenolides show lower rates and burden of infection (Gowler et al. 2015; Lefèvre et al. 2012; Sternberg et al. 2012; Hoogshagen et al. 2024).

In this study, we examine the relative contributions of host family (genetic effect), larval food plant (environment) and their interaction on susceptibility and resistance in the monarch to OE. We used two species of milkweed that differ in their concentrations of cardenolide toxins but are similar in nutrient composition: *A. curassavica* has up to 95 times higher total cardenolide concentrations than *A. incarnata* (Roeske et al. 1976; Malcolm 1991; Tao et al. 2016; Tan et al. 2019). We expected to find significant genetic variation in infection outcomes and that monarch families raised on *A. curassavica* , regardless of their genotype, would be less likely to become infected and experience lower infection burden and longer lifespan due to the anti‐parasitic properties of the high cardenolide plant species.

## 2 Materials and Methods

### 2.1 Experimental Design

Monarchs used in this study were grand progeny of wild adults caught in St. Marks in Florida, USA in October 2020 and overwintered in the laboratory at Emory University, Atlanta, Georgia, USA. In late March 2021, F1 adults were allowed to mate in mesh cages in a growth chamber kept at 26°C, 16L: 8D light cycle, to create 31 distinct non‐inbred full‐sib families. Mated females laid eggs on *A. incarnata* plants and upon hatching, we randomly selected caterpillars to either *A. incarnata* or *A. curassavica* treatment and placed the hatchlings on a new respective plant. At the second instar larval stage, we individually inoculated caterpillars with *O. elektroscirrha* parasites: caterpillars were fed a 0.5 cm^2^ leaf disk of *A. incarnata* or *A. curassavica* with 1 manually deposited spore in a Petri dish. Upon complete consumption of the leaf disk, caterpillars were transferred to individual plants of either *A. incaranata* or *A. curassavica* covered with a transparent plastic tube (4 in. diameter × 24 in. height; 10.16 cm × 61 cm) and capped with netting. We used 15 replicate caterpillars per family in each of the two treatments. Plants used in this experiment were grown in the greenhouse from seeds at 26°C, 16L: 8D light cycle, and were similar in height 20–24 in. (50.8–61 cm) with two stalks. Caterpillars were monitored daily and provided fresh plants as needed to assure food supply was *ad libitum*. Upon pupation we glued each pupa to the lid of an individual 16 oz. (473 mL) SOLO cup. Once adult butterflies emerged, we placed them in glassine envelopes and kept them in an incubator at 12°C and checked them daily until death. We quantified adult lifespan as the number of days from emergence to death. Work by de Roode et al. (2009) shows that this measure of lifespan correlates with lifespan and fecundity under more natural conditions. Upon death, we measured the length of the right wing of each adult.

### 2.2 Infection Assessment

We obtained pupal infection scores by visually inspecting the pupa on the day before expected emergence following previously established protocols (de Roode et al. 2009). Pupal infection score ranks the level of dark coloration on the pupal casing on a scale of 0–5, with a score of 0 being no visible infection and a score of 5 being very severe visible infection. Monarchs with scores of 0 were classified as uninfected and those with scores higher than 0 were classified as infected. For a subset of butterflies (*n* = 592 individuals), we assessed parasite spore load after death by vortexing the bodies in 5 mL of water and counting the number of spores in 10 μL of the suspension using a hemocytometer slide (de Roode et al. 2007). We quantified the relationship between the pupal infection score and parasite spore load using a simple linear regression to derive an estimated spore load for the remainder of individuals: [log_10_(spore load) = 5.06 + 1.73 × (pupal score); *F*_1,91_ = 47.03, *p* < 0.001, *R* ^2^ = 0.34]. This approach yielded five levels of estimated parasite spore load (5.06 × 10^5^, 5.58 × 10^5^, 5.88 × 10^5^, 6.10 × 10^5^, 6.26 × 10^5^).

### 2.3 Statistical Analysis

We used R programming for all analyses (R Core Team 2025). We conducted genotype × environment (G × E) interaction analysis using mixed‐effects models following Dia et al. (2017). This approach consists of running a series of models that partition total phenotypic variance into components attributable to genotype, environment, and their interaction, and testing the significance of each component via likelihood ratio tests comparing full models versus reduced null models with each effect removed (Table 1). The first model included family, plant species, and their interaction as random effects to estimate variance components (Table 1, Model 1). The second model included family as a fixed effect and plant species and the interaction term as random effects (Table 1, Model 2). The third model included plant species and the interaction term as random effects only (Table 1, Model 2_o_). The fourth model included plant species as a fixed effect and family and the interaction as random effects (Table 1, Model 3). The fifth model included family and the interaction as random effects only (Table 1, Model 3_o_). The sixth model was identical to the first but with the family by plant species interaction removed (Table 1, Model 4). Next, the significance of each effect was assessed using likelihood ratio tests comparing pairs of models with and without the term of interest, family effect: Model 2 versus Model 2_o_; plant species effect: Model 3 versus Model 3_o_; G × E interaction: Model 1 versus Model 4. For each comparison we computed a chi‐square statistic with degrees of freedom equal to the difference in the number of parameters between models. A total of six models were fit per trait (infection status, infected adult spore load, infected adult lifespan, and infected adult wing length). For model formulas see Table 1.

**TABLE 1:** Series of generalized linear mixed models for family and plant species (G × E) interaction analyses following the framework by Dia et al. (2017).

Model	Fixed effects	Random effects	df	Model formula
Model 1	Intercept	Family, Plant species, G × E, Inoculation date	4	Trait ~1 + (1|Family) + (1|Plant species) + (1|Family × Plant species) + (1| Inoculation date)
Model 2	Intercept, Family	Plant species, G × E, Inoculation date	33	Trait ~1 + Family + (1|Plant species) + (1|Family × Plant species) + (1|Inoculation date)
Model 2_o_	Intercept	Plant species, G × E, Inoculation date	3	Trait ~1 + (1|Plant_species) + (1|Family × Plant species) + (1|Inoculation date)
Model 3	Intercept, Plant species	Family, G × E, Inoculation date	4	Trait ~1 + Plant species + (1|Family) + (1|Family × Plant species) + (1|Inoculation date)
Model 3_o_	Intercept	Family, G × E, Inoculation date	3	Trait ~1 + (1|Family) + (1|Family × Plant species) + (1|Inoculation date)
Model 4	Intercept	Family, Plant species, Inoculation date	3	Trait ~1 + (1|Family) + (1|Plant species) + (1|Inoculation date)

*Note:* Trait in the model formula represents characteristics examined in the study: Infection status (0/1), infected adult spore load (infection burden), infected adult lifespan, infected adult wing length. Model comparisons include (1) Family effect: Model 2 versus Model 2_o_, (2) Plant species effect: Model 3 versus Model 3_o_, and (3) G × E interaction: Model 1 versus Model 4.

We used generalized linear mixed models (GLMM) for infection status, with 0 = uninfected and 1 = infected as the response variable and binomial distribution with logit link function. For spore load, lifespan of infected monarchs, and infected adult wing length, we fit linear mixed models (LMMs). We included inoculation date as a random effect in all models to account for temporal variation (inoculations were carried out over a span of 8 days). For significant effects of plant species, we conducted follow up analysis: post hoc pairwise comparisons using estimated marginal means with Tukey adjustments for multiple comparisons. We did not conduct post hoc analysis on family comparisons given that family level comparisons are not meaningful in this context.

## 3 Results

### 3.1 General Results

Of the 921 caterpillars with which we initiated the study, 19 caterpillars perished in the *A. incarnata* and 18 in the *A. curassavica* treatments prior to adult eclosion due to unknown causes, yielding 96% survival to adulthood. Survival to adulthood varied between 70 and 100% across families. 39.8% of caterpillars (*n* = 442) became infected in *A. curassavica* treatment, while in the *A. incarnata* treatment 47.5% of caterpillars (*n* = 442) became infected. The adult lifespan of infected monarchs ranged from 8 to 35 days (mean = 20.73, SE = 0.28, *n* = 386). Infected monarchs raised on *A. curassavica* lived an average of 23.5 days (*n* = 176) versus 18.5 days on *A. incarnata* (*n* = 210). The wing length of infected monarchs varied between 49 and 57.5 mm (mean = 53.34, SE = 0.08, *n* = 382).

### 3.2 G x E Analysis Results

**FIGURE 1:**
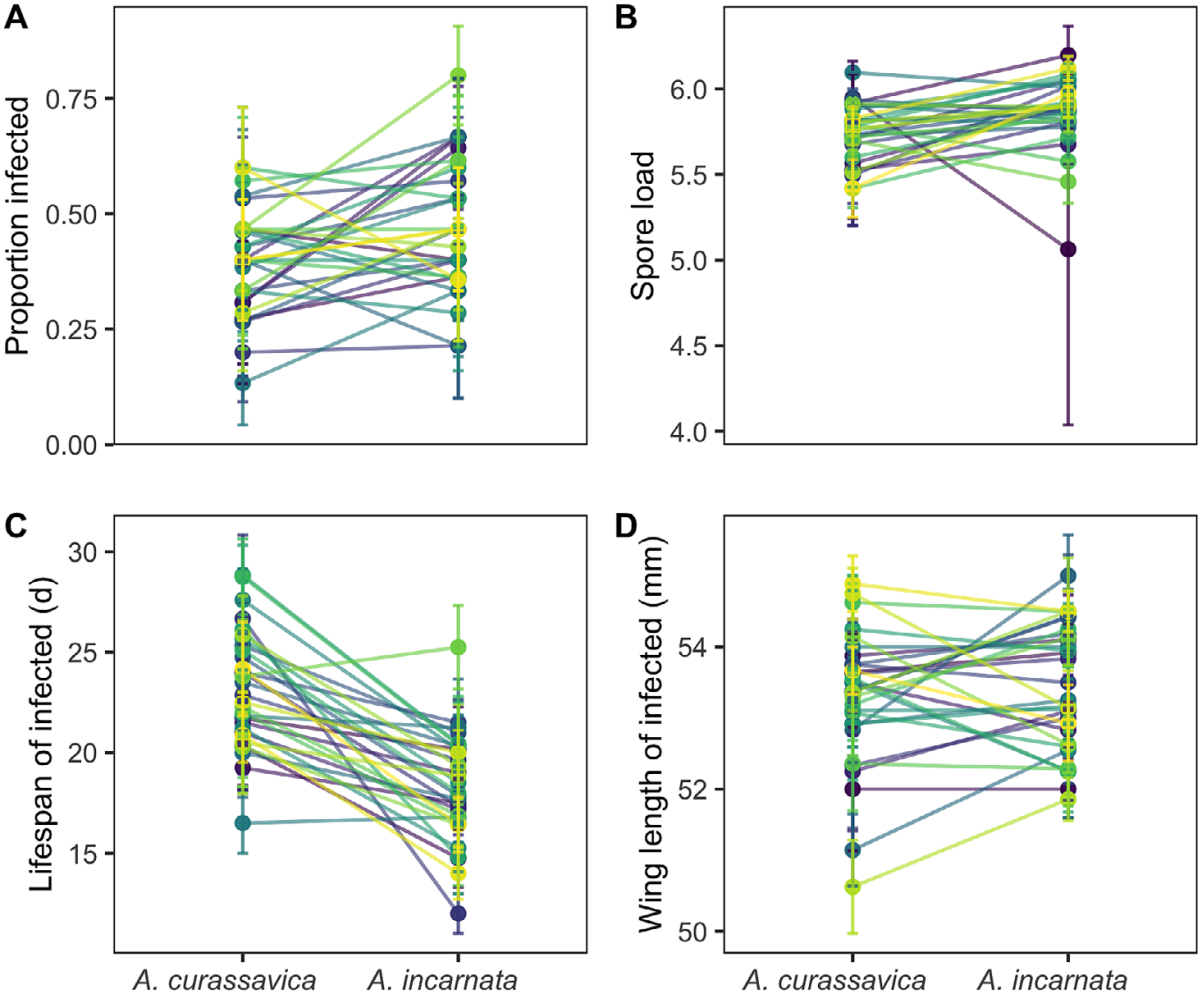
Reaction norms across two milkweed species, *A. curassavica* and *A. incarnata* , for four traits measured in 31 full‐sib families: (A) proportion of infected monarchs, (B) spore load of infected adults, (C) lifespan of infected adults, and (D) wing length of infected adults. Points represent family means, with error bars showing ±1 SE within family. Lines connect family means across the two plant species. Different colors represent different families.

To understand the role of environment and genotype on monarch infection outcomes, we used genotype × environment (G × E) interaction analysis. We found no G × E interaction between plant species (environment) and family (genetic) on any of the measured traits: infection status, spore load, infected adult lifespan, or infected adult wing length (Table 2, Figure 1). For all four traits, we conducted additional follow up analysis to determine whether G × E non‐significance reflected limited statistical power. We used variance components from Model 1 to compute the ratio of G × E variance to within‐family residual variance for each trait. Results indicate that within‐family residual variance accounted for most of the total variance across all four traits, with G × E variance representing at most 5.2% of total variance (see Tables S1, S2).

**TABLE 2:** Variance components and descriptive statistics from family and plant species (G × E) interaction analysis (models specified in Table 1) for four traits: Infection status, infected adult spore load, infected adult lifespan, and infected adult wing length.

Trait	Family (G)	Plant (E)	G × E	Error	Total	Mean	CV (%)	MSE
Infection status (0/1)	0.02**	0.02	0	3.29	3.33	0.44	113.65	3.29
Infected adult spore load	0	0.01*	0	0.18	0.19	5.80	7.52	0.18
Infected adult lifespan	2.13**	12.09***	0.87	21.55	36.65	20.73	26.67	21.55
Infected adult wing length	0.37***	0	0.12	1.86	2.35	53.34	2.86	1.86

*Note:* Total is the sum of all variance components, mean is the average value of the trait, CV is the coefficient of variation, and MSE is the mean squared error. Bolded variance components indicate significant effects with stars representing *p*‐values: ***p ≤ 0.001, **p ≤ 0.01, *p ≤ 0.05. For full model outputs see Tables S3–S6.

**FIGURE 2:**
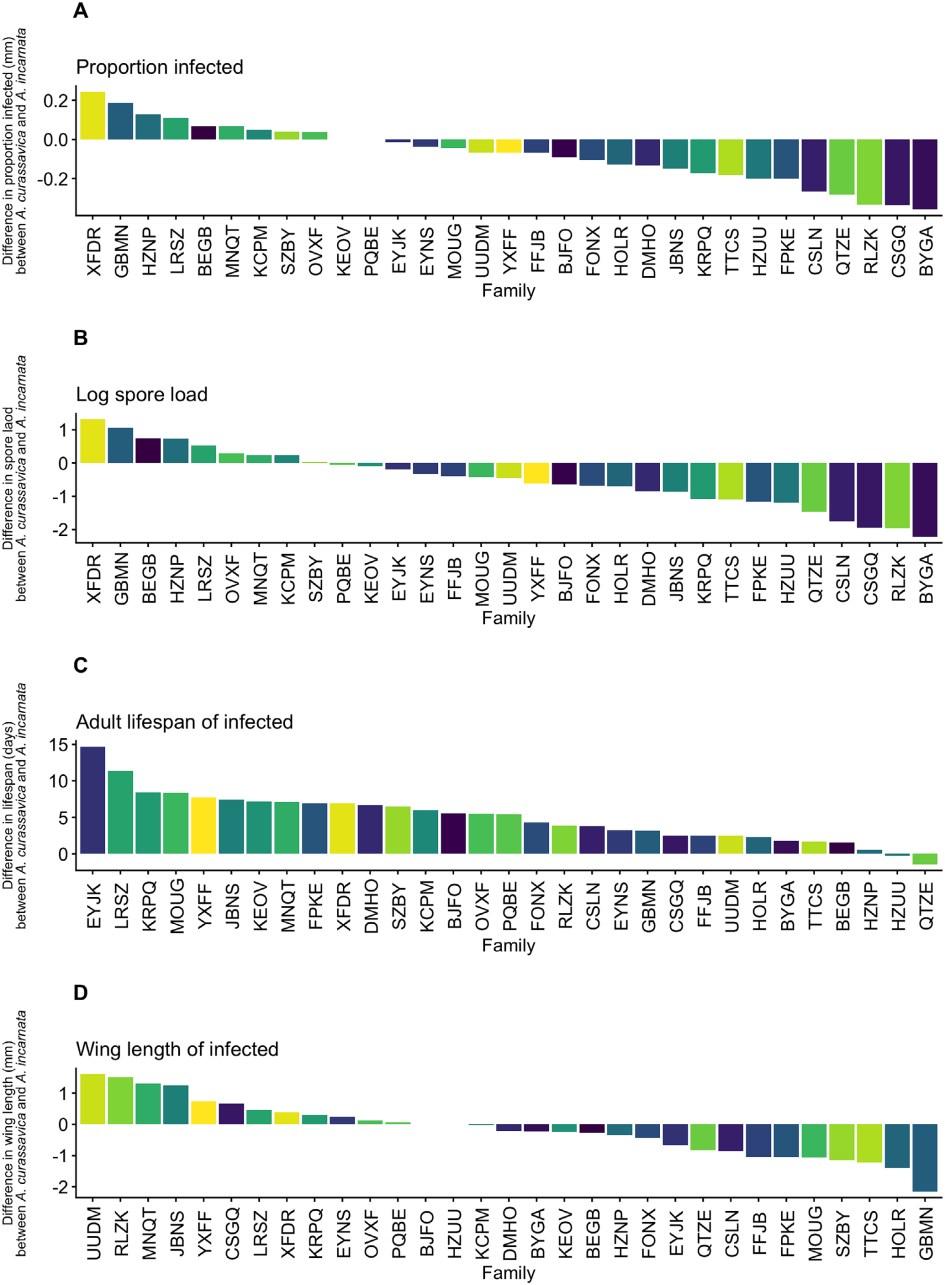
Difference between monarchs reared on *A. curassavica* and *A. incarnata* in (A) proportion of infected monarchs among monarch full‐sib families, (B) average spore loads of infected monarchs in each family, (C) lifespan of infected adults, and (D) wing length of infected adults. Different colors represent different families. Positive values (above zero) indicate higher values for *A. curassavica* and negative values (below zero) indicate higher values for *A. incarnata* .

G × E analyses showed an effect of plant species on infection status, but no effect of family on infection status (Table 2, Figure 2A). Follow up analysis indicated a greater number of monarchs were infected when reared on *A. incarnata* compared to *A. curassavica* (*t* = 9.12, *p* < 0.001). Plant species, but not family, explained variation in spore load of infected monarchs, with follow up analysis indicating lower spore load on monarchs reared on *A. curassavica* compared to *A. incarnata* (*t* = −2.56, *p* = 0.02; Table 2, Figure 2B). G × E analyses indicated significant effects of plant species and family on lifespan of infected adults (Table 2, Figure 2C). Follow up analysis showed infected monarchs lived longer when raised on *A. curassavica* than on *A. incarnata* (*t* = 9.16, df = 28.8, *p* < 0.001). Finally, G × E analyses indicated that family but not plant species impacted infected adult wing length (Table 2, Figure 2D).

## 4 Discussion

In this study we asked whether host genetics and environment (here: larval food plant species) interact and lead to differences in infection outcomes in a specialist herbivore, the monarch. We found no genotype by plant environment interactions across various measures of infection, including infection status, infection load, adult lifespan, and adult size. However, we noted main effects of plant species on infection status and load, and adult lifespan. Also, we observed main effects of host family on adult lifespan and size of infected adults, suggesting that genetic and environmental effects operate independently. Few other studies report a lack of genotype by environmental interactions on infection measures (e.g., Karvonen et al. 2013). For instance, Gimmi and Vorburger (2021) found that strong genotype‐by‐genotype (G × G) interaction between a black bean aphid ( *Aphis fabae* ) endosymbiont and a parasitoid *Lysiphlebus fabarum* were maintained across different host plants, with no evidence for G × G × E interactions despite main effects of plant species on parasitism rates. Together our work supports the idea that in some systems G × E interactions may be trait or context‐specific (Pour‐Aboughadareh et al. 2022). We decided to examine milkweed species as an environmental factor because it has been shown to importantly alter host susceptibility to infection. However many other environmental conditions, such as temperature (Ragonese et al. 2025) and host density (Majewska et al. 2019), might interact with genetic factors in the monarch—OE system and warrant further investigation.

We found a strong effect of plant species on infection status, with monarchs being more susceptible to infection when reared on *A. incarnata* : the proportion of infected monarchs was higher for monarch larvae fed *A. incarnata* compared to *A. curassavica* . This work confirms previous findings showing the same patterns (e.g., Tan et al. 2019) and is consistent with our expectations based on prior work showing high concentrations of cardenolides in *A. curassavica* reduce infection probability (Gowler et al. 2015; Lefèvre et al. 2012; Sternberg et al. 2012; Hoogshagen et al. 2024). Similarly, we found the expected relationship between lower parasite load in monarchs fed *A. curassavica* compared to *A. incarnata* due to the *A. curassavica'*s high cardenolide concentrations suppressing parasite growth within the host (Hoogshagen et al. 2024). Diet influencing resistance and pathogen load have been described in other insect systems, including fruit flies (*Drosophila*), cabbage whites ( *Pieris rapae* ), African cotton leafworm (*Spodoptera littoralis*), and mosquitoes (*Anopheles sephensi*) (Howick and Lazzaro 2014; Ghosh et al. 2023; Koella and Sorense 2002), indicating that diet plays a key role in insect infection dynamics.

Interestingly, both plant species and host genetics (as measured through variation in full‐sib families) influenced infected monarchs' lifespan, with monarchs raised on *A. curassavica* living longer than those raised on *A. incarnata* . Lifespan is a fitness measure that integrates many physiological processes, including molecular damage (e.g., oxidative stress) and the efficiency of repair mechanisms (Bonsall 2006; Tian et al. 2017; O'neill et al. 2019). Genetic effects might be operating through immune responses that function similarly regardless of plant chemistry. Therefore, our result suggests that host diet effects operate through plant secondary compounds that influence host condition and physiology. Given that high cardenolides in larval diet can decrease OE growth within the host (de Roode et al. 2008; Hoogshagen et al. 2024), likely the damage the parasite causes is also reduced, yielding longer lifespan in adults. In addition, a number of genes are upregulated and downregulated in larvae fed *A. curassavica* , some of which allow the larvae to metabolize the toxin, while others remain uncharacterized (Tan et al. 2019), but may play a role in physiological processes pivotal for longevity in parasitized monarchs. Further work is needed to enhance our understanding of the mechanisms responsible for longer adult life in monarch fed *A. curassavica* as larvae.

Finally, monarch family but not plant species was a strong predictor of monarch wing size of infected adults. While prior studies on monarchs indicate that size is influenced by the amount of milkweed consumed by the larva (Alaidrous et al. 2022; Cibotti et al. 2025), our work indicates that when larval food plant is plentiful, morphology is more dictated by host genetics. The wing size being solely determined by family suggests that different developmental mechanisms govern morphology compared to immune responses and resistance to parasite invasion.

In conclusion, we have demonstrated that in monarchs, larval food plants significantly influence interactions with a protozoan parasite, but genetic variation across families does not interact with this environmental factor to modify infection outcomes. Our results suggest that both environment (in terms of host plant species) and monarch genetics (as measured through variation among full‐sib families) independently shape infection dynamics and fitness consequences. Accordingly, future studies should consider milkweed plants that vary in nutritional quality (i.e., field relevant differences in carbon to nitrogen composition), additional milkweed species, and other interactions, including those between host genotype, temperature, parasite genotype, and the milkweed species.

## Author Contributions

**Ania A. Majewska:** conceptualization (equal), data curation (equal), formal analysis (equal), investigation (equal), methodology (equal), project administration (equal), visualization (equal), writing – original draft (equal), writing – review and editing (lead). **Grace Chlebove:** investigation (equal), writing – original draft (equal). **Jacobus C. de Roode:** conceptualization (equal), funding acquisition (equal), investigation (equal), project administration (equal), resources (equal), supervision (equal), validation (equal), writing – review and editing (equal).

## Funding

This work was supported by the National Institute of General Medical Sciences, 5K12GM000680‐19. Directorate for Biological Sciences, DEB‐1754431.

## Conflicts of Interest

The authors declare no conflicts of interest.

## Supporting information


**Table S1:** Sample sizes (*n*) and coefficient of variation of *n* across families.
**Table S2:** Variance components estimated from a model in which family, plant species, and their interaction were included as random effects (main text Table 1, Model 1).
**Table S3:** Full model outputs for infection status.
**Table S4:** Full model outputs for spore load.
**Table S5:** Full model outputs for adult lifespan.
**Table S6:** Full model outputs for wing length.

## Data Availability

Data to reproduce the analyses are provided on Figshare at https://doi.org/10.6084/m9.figshare.31069678.
