# Supplementary material for: Environment and Host Genetics Drive Infection Outcomes in a Butterfly Host, but Do Not Interact With Each Other
Source: Ecol Evol. 2026 Aug 3;16(8):e74075. doi: 10.1002/ece3.74075 (PMC13433141; doi:10.1002/ece3.74075)
Supplement: Supplementary file 1 [file ECE3-16-e74075-s001.docx]

**Supporting Information**

To accompany:

*Environment and host genetics drive infection outcomes in a butterfly host, but do not interact with each other*

Ania A. Majewska, Grace Chlebove, Jacobus de Roode

**Table S1.** Sample sizes (*n*) and coefficient of variation of *n* across families.

| **Plant species** | **Mean *n* per family** | **SD** | **Min *n*** | **Max *n*** | **CV (%)** |
| --- | --- | --- | --- | --- | --- |
| *A. curassavica* | 14.3 | 1.3 | 10 | 15 | 8.9 |
| *A. incarnata* | 14.3 | 1.2 | 11 | 15 | 8.1 |

**Table S2.** Variance components estimated from a model in which family, plant species, and their interaction were included as random effects (main text Table 1, Model 1), partitioning total phenotypic variance into family (genetic, G), plant species (environmental, E), GxE interaction, and residual components. Residual variance is within-family residual variance. For infection (binary 0/1) within-family residual variance is fixed at π²/3 ≈ 3.29 (logit scale). Ratio values of GxE variance: residual variance close to zero indicate that interaction variance is small relative to within-family variation*.*

| **Trait** | **Family variance** | **Plant species variance** | **GxE variance** | **Residual variance** | **Total variance** | **Family variance %** | **Plant species variance %** | **GxE interaction variance %** | **Ratio GxE variance: residual variance** |
| --- | --- | --- | --- | --- | --- | --- | --- | --- | --- |
| Infection | 0.02 | 0.02 | 0.00 | 3.29 | 3.33 | 0.7 | 0.5 | 0.0 | 0.00 |
| Adult longevity | 2.13 | 12.09 | 0.87 | 21.55 | 36.65 | 5.8 | 33.0 | 2.4 | 0.04 |
| Wing length | 0.37 | 0.00 | 0.12 | 1.86 | 2.35 | 15.7 | 0.0 | 5.2 | 0.07 |
| Spore load | 0.00 | 0.01 | 0.00 | 0.18 | 0.19 | 0.0 | 3.2 | 2.1 | 0.02 |

**Table S3.** Full model outputs for infection status. a) Model 1, including family, plant species, and their interaction as random effects. b) Model 2, with family as a fixed effect and plant species and the interaction as random effects. c) Model 2₀, the corresponding null model with family removed entirely. d) Model 3, with plant species as a fixed effect and family and the interaction as random effects. e) Model 3₀, the corresponding null model with plant species removed entirely. f) Model 4, identical to Model 1 but with the family by plant species interaction removed.

**a.**

|  | **Model 1 Infection (0/1)** | | | |
| --- | --- | --- | --- | --- |
| *Fixed effects* | *Odds Ratios* | *SE* | *Statistic* | *p* |
| (Intercept) | 0.77 | 0.10 | -2.09 | **0.037** |
| **Random Effects** | | | | |
| Residual variance | 3.29 | | | |
| Family x Plant species | 0.00 | | | |
| Family | 0.02 | | | |
| Inoculation date | 0.01 | | | |
| Plant species | 0.02 | | | |

**b.**

|  | **Model 2 Infection (0/1)** | | | |
| --- | --- | --- | --- | --- |
| *Fixed effects* | *Odds Ratios* | *SE* | *Statistic* | *p* |
| (Intercept) | 0.76 | 0.29 | -0.71 | 0.479 |
| Family [BJFO] | 0.61 | 0.36 | -0.84 | 0.399 |
| Family [BYGA] | 1.30 | 0.69 | 0.50 | 0.620 |
| Family [CSGQ] | 1.21 | 0.65 | 0.36 | 0.721 |
| Family [CSLN] | 1.50 | 0.78 | 0.78 | 0.438 |
| Family [DMHO] | 0.65 | 0.35 | -0.80 | 0.425 |
| Family [EYJK] | 0.34 | 0.20 | -1.83 | 0.068 |
| Family [EYNS] | 1.62 | 0.85 | 0.92 | 0.360 |
| Family [FFJB] | 0.76 | 0.40 | -0.53 | 0.597 |
| Family [FONX] | 1.22 | 0.64 | 0.37 | 0.708 |
| Family [FPKE] | 0.76 | 0.40 | -0.53 | 0.597 |
| Family [GBMN] | 0.59 | 0.32 | -0.97 | 0.332 |
| Family [HOLR] | 2.01 | 1.08 | 1.31 | 0.191 |
| Family [HZNP] | 0.87 | 0.48 | -0.24 | 0.808 |
| Family [HZUU] | 0.40 | 0.23 | -1.63 | 0.104 |
| Family [JBNS] | 1.13 | 0.60 | 0.22 | 0.823 |
| Family [KCPM] | 0.59 | 0.32 | -0.97 | 0.332 |
| Family [KEOV] | 0.87 | 0.46 | -0.26 | 0.792 |
| Family [KRPQ] | 1.40 | 0.73 | 0.64 | 0.523 |
| Family [LRSZ] | 0.93 | 0.49 | -0.15 | 0.884 |
| Family [MNQT] | 1.71 | 0.89 | 1.03 | 0.302 |
| Family [MOUG] | 1.91 | 1.03 | 1.21 | 0.228 |
| Family [OVXF] | 0.80 | 0.47 | -0.38 | 0.701 |
| Family [PQBE] | 1.14 | 0.60 | 0.26 | 0.795 |
| Family [QTZE] | 1.14 | 0.60 | 0.25 | 0.802 |
| Family [RLZK] | 2.27 | 1.20 | 1.55 | 0.122 |
| Family [SZBY] | 1.07 | 0.56 | 0.12 | 0.903 |
| Family [TTCS] | 0.80 | 0.42 | -0.43 | 0.667 |
| Family [UUDM] | 1.00 | 0.52 | -0.00 | 0.999 |
| Family [XFDR] | 1.23 | 0.64 | 0.39 | 0.698 |
| Family [YXFF] | 1.00 | 0.52 | -0.00 | 0.999 |
| **Random Effects** | | | | |
| Plant species | 0.02 | | | |
| Family x Plant species | 0.00 | | | |
| Inoculation date | 0.00 | | | |
| *Residual variance* | 3.29 | | | |

**c.**

|  | **Model 2 (null) Infection (0/1)** | | | |
| --- | --- | --- | --- | --- |
| *Fixed effects* | *Odds Ratios* | *SE* | *Statistic* | *p* |
| (Intercept) | 0.77 | 0.10 | -2.09 | **0.037** |
| **Random Effects** | | | | |
| Plant species | 0.02 | | | |
| Family x Plant species | 0.00 | | | |
| Inoculation date | 0.02 | | | |
| *Residual variance* | 3.29 | | | |

**d.**

|  | **Model 3 Infection (0/1)** | | | |
| --- | --- | --- | --- | --- |
| *Fixed effects* | *Odds Ratios* | *SE* | *Statistic* | *p* |
| (Intercept) | 0.66 | 0.07 | -3.92 | **<0.001** |
| Plant species [inc] | 1.37 | 0.19 | 2.30 | **0.021** |
| **Random Effects** | | | | |
| Family | 0.02 | | | |
| Family x Plant species | 0.00 | | | |
| Inoculation date | 0.01 | | | |
| *Residual variance* | 3.29 | | | |

**e.**

|  | **Model 3 (null) Infection (0/1)** | | | |
| --- | --- | --- | --- | --- |
| *Fixed effects* | *Odds Ratios* | *SE* | *Statistic* | *p* |
| (Intercept) | 0.77 | 0.06 | -3.19 | **0.001** |
| **Random Effects** | | | | |
| Family | 0.02 | | | |
| Family x Plant species | 0.00 | | | |
| Inoculation date | 0.01 | | | |
| *Residual variance* | 3.29 | | | |

**f.**

|  | **Model 4 Infection (0/1)** | | | |
| --- | --- | --- | --- | --- |
| *Fixed effects* | *Odds Ratios* | *SE* | *Statistic* | *p* |
| (Intercept) | 0.77 | 0.10 | -2.08 | **0.037** |
| **Random Effects** | | | | |
| Plant species | 0.02 | | | |
| Family | 0.02 | | | |
| Inoculation date | 0.01 | | | |
| *Residual variance* | 3.29 | | | |

**Table S4.** Full model outputs for spore load. a) Model 1, including family, plant species, and their interaction as random effects. b) Model 2, with family as a fixed effect and plant species and the interaction as random effects. c) Model 2₀, the corresponding null model with family removed entirely. d) Model 3, with plant species as a fixed effect and family and the interaction as random effects. e) Model 3₀, the corresponding null model with plant species removed entirely. f) Model 4, identical to Model 1 but with the family by plant species interaction removed.

**a.**

|  | **Model 1 – Spore load** | | | | |
| --- | --- | --- | --- | --- | --- |
| *Fixed effects* | *Estimate* | *SE* | *Statistic* | *df* | *p* |
| (Intercept) | 5.79 | 0.06 | 96.02 | 380 | **<0.001** |
| **Random Effects** | | | | | |
| Plant species | 0.01 | | | | |
| Family | 0.00 | | | | |
| Family x Plant species | 0.00 | | | | |
| Inoculation date | 0.00 | | | | |
| *Residual variance* | 0.18 | | | | |

**b.**

|  | **Model 2 - Spore load** | | | | |
| --- | --- | --- | --- | --- | --- |
| *Fixed effects* | *Estimate* | *SE* | *Statistic* | *df* | *p* |
| (Intercept) | 5.54 | 0.14 | 38.66 | 351 | **<0.001** |
| Family [BJFO] | 0.53 | 0.22 | 2.45 | 351 | **0.015** |
| Family [BYGA] | 0.24 | 0.19 | 1.30 | 351 | 0.196 |
| Family [CSGQ] | 0.07 | 0.19 | 0.39 | 351 | 0.693 |
| Family [CSLN] | 0.37 | 0.18 | 2.04 | 351 | **0.042** |
| Family [DMHO] | 0.14 | 0.20 | 0.72 | 351 | 0.470 |
| Family [EYJK] | 0.22 | 0.23 | 0.97 | 351 | 0.331 |
| Family [EYNS] | 0.24 | 0.18 | 1.33 | 351 | 0.185 |
| Family [FFJB] | 0.36 | 0.19 | 1.84 | 351 | 0.066 |
| Family [FONX] | 0.28 | 0.18 | 1.50 | 351 | 0.134 |
| Family [FPKE] | 0.35 | 0.19 | 1.78 | 351 | 0.075 |
| Family [GBMN] | 0.23 | 0.20 | 1.11 | 351 | 0.269 |
| Family [HOLR] | 0.32 | 0.18 | 1.78 | 351 | 0.076 |
| Family [HZNP] | 0.31 | 0.20 | 1.55 | 351 | 0.123 |
| Family [HZUU] | 0.48 | 0.22 | 2.21 | 351 | **0.028** |
| Family [JBNS] | 0.27 | 0.19 | 1.42 | 351 | 0.157 |
| Family [KCPM] | 0.42 | 0.20 | 2.08 | 351 | **0.038** |
| Family [KEOV] | 0.40 | 0.19 | 2.12 | 351 | **0.035** |
| Family [KRPQ] | 0.31 | 0.18 | 1.69 | 351 | 0.092 |
| Family [LRSZ] | 0.38 | 0.19 | 2.02 | 351 | **0.044** |
| Family [MNQT] | 0.19 | 0.18 | 1.07 | 351 | 0.285 |
| Family [MOUG] | 0.03 | 0.18 | 0.19 | 351 | 0.853 |
| Family [OVXF] | 0.15 | 0.21 | 0.72 | 351 | 0.473 |
| Family [PQBE] | 0.24 | 0.18 | 1.28 | 351 | 0.202 |
| Family [QTZE] | 0.01 | 0.19 | 0.06 | 351 | 0.955 |
| Family [RLZK] | 0.36 | 0.18 | 2.03 | 351 | **0.043** |
| Family [SZBY] | 0.17 | 0.19 | 0.93 | 351 | 0.352 |
| Family [TTCS] | 0.32 | 0.19 | 1.62 | 351 | 0.105 |
| Family [UUDM] | 0.30 | 0.19 | 1.59 | 351 | 0.113 |
| Family [XFDR] | 0.42 | 0.18 | 2.25 | 351 | **0.025** |
| Family [YXFF] | 0.17 | 0.19 | 0.93 | 351 | 0.352 |
| **Random Effects** | | | | | |
| Plant species | 0.01 | | | | |
| Family x Plant species | 0.01 | | | | |
| Inoculation date | 0.00 | | | | |
| *Residual variance* | 0.18 | | | | |

**c.**

|  | **Model 2 (null) – Spore load** | | | | |
| --- | --- | --- | --- | --- | --- |
| *Fixed effects* | *Estimate* | *SE* | *Statistic* | *df* | *p* |
| (Intercept) | 5.79 | 0.06 | 96.06 | 381 | **<0.001** |
| **Random Effects** | | | | | |
| Plant species | 0.01 | | | | |
| Family x Plant species | 0.00 | | | | |
| Inoculation date | 0.00 | | | | |
| *Residual variance* | 0.18 | | | | |

**d.**

|  | **Model 3 – Spore load** | | | | |
| --- | --- | --- | --- | --- | --- |
| *Fixed effects* | *Estimate* | *SE* | *Statistic* | *df* | *p* |
| (Intercept) | 5.73 | 0.03 | 166.93 | 380 | **<0.001** |
| Plant species [inc] | 0.12 | 0.05 | 2.58 | 380 | **0.010** |
| **Random Effects** | | | | | |
| Family | 0.00 | | | | |
| Family x Plant species | 0.00 | | | | |
| Inoculation date | 0.00 | | | | |
| *Residual variance* | 0.18 | | | | |

**e.**

|  | **Model 3 (null) – Spore load** | | | | |
| --- | --- | --- | --- | --- | --- |
| *Fixed effects* | *Estimate* | *SE* | *Statistic* | *df* | *p* |
| (Intercept) | 5.80 | 0.02 | 236.52 | 381 | **<0.001** |
| **Random Effects** | | | | | |
| Family | 0.00 | | | | |
| Family x Plant species | 0.01 | | | | |
| Inoculation date | 0.00 | | | | |
| *Residual variance* | 0.18 | | | | |

**f.**

|  | **Model 4 – Spore load** | | | | |
| --- | --- | --- | --- | --- | --- |
| *Fixed effects* | *Estimate* | *SE* | *Statistic* | *df* | *p* |
| (Intercept) | 5.79 | 0.06 | 94.82 | 381 | **<0.001** |
| **Random Effects** | | | | | |
| Plant species | 0.01 | | | | |
| Family | 0.00 | | | | |
| Inoculation date | 0.00 | | | | |
| *Residual variance* | 0.19 | | | | |

**Table S5.** Full model outputs for adult lifespan. a) Model 1, including family, plant species, and their interaction as random effects. b) Model 2, with family as a fixed effect and plant species and the interaction as random effects. c) Model 2₀, the corresponding null model with family removed entirely. d) Model 3, with plant species as a fixed effect and family and the interaction as random effects. e) Model 3₀, the corresponding null model with plant species removed entirely. f) Model 4, identical to Model 1 but with the family by plant species interaction removed.

**a.**

|  | **Model 1 - Adult Lifespan** | | | | |
| --- | --- | --- | --- | --- | --- |
| *Fixed effects* | *Estimate* | *SE* | *Statistic* | *df* | *p* |
| (Intercept) | 20.87 | 2.49 | 8.39 | 380 | **<0.001** |
| **Random Effects** | | | | | |
| Plant species | 12.11 | | | | |
| Family | 2.13 | | | | |
| Family x Plant species | 0.87 | | | | |
| Inoculation date | 0.00 | | | | |
| *Residual variance* | 21.55 | | | | |

**b**.

|  | **Model 2 - Adult Lifespan** | | | | |
| --- | --- | --- | --- | --- | --- |
| *Fixed effects* | *Estimate* | *SE* | *Statistic* | *df* | *p* |
| (Intercept) | 20.85 | 2.87 | 7.27 | 351 | **<0.001** |
| Family [BJFO] | -3.35 | 2.42 | -1.38 | 351 | 0.168 |
| Family [BYGA] | -1.96 | 2.10 | -0.93 | 351 | 0.353 |
| Family [CSGQ] | -0.24 | 2.13 | -0.11 | 351 | 0.909 |
| Family [CSLN] | -1.65 | 2.04 | -0.81 | 351 | 0.420 |
| Family [DMHO] | -0.32 | 2.23 | -0.14 | 351 | 0.885 |
| Family [EYJK] | -1.51 | 2.53 | -0.60 | 351 | 0.550 |
| Family [EYNS] | 0.40 | 2.04 | 0.20 | 351 | 0.843 |
| Family [FFJB] | 1.99 | 2.18 | 0.91 | 351 | 0.363 |
| Family [FONX] | 2.35 | 2.08 | 1.13 | 351 | 0.260 |
| Family [FPKE] | 0.24 | 2.19 | 0.11 | 351 | 0.912 |
| Family [GBMN] | 0.84 | 2.29 | 0.37 | 351 | 0.715 |
| Family [HOLR] | -1.84 | 2.02 | -0.91 | 351 | 0.363 |
| Family [HZNP] | 0.36 | 2.23 | 0.16 | 351 | 0.873 |
| Family [HZUU] | -3.23 | 2.44 | -1.32 | 351 | 0.187 |
| Family [JBNS] | 2.79 | 2.12 | 1.32 | 351 | 0.188 |
| Family [KCPM] | -2.57 | 2.28 | -1.13 | 351 | 0.260 |
| Family [KEOV] | 1.24 | 2.14 | 0.58 | 351 | 0.564 |
| Family [KRPQ] | 3.54 | 2.06 | 1.72 | 351 | 0.087 |
| Family [LRSZ] | 0.04 | 2.15 | 0.02 | 351 | 0.986 |
| Family [MNQT] | 0.76 | 2.02 | 0.37 | 351 | 0.708 |
| Family [MOUG] | 3.72 | 2.04 | 1.83 | 351 | 0.069 |
| Family [OVXF] | -1.60 | 2.34 | -0.68 | 351 | 0.496 |
| Family [PQBE] | -1.27 | 2.08 | -0.61 | 351 | 0.541 |
| Family [QTZE] | 4.23 | 2.12 | 2.00 | 351 | **0.047** |
| Family [RLZK] | -2.41 | 1.99 | -1.21 | 351 | 0.228 |
| Family [SZBY] | 1.80 | 2.11 | 0.85 | 351 | 0.395 |
| Family [TTCS] | -0.82 | 2.19 | -0.38 | 351 | 0.708 |
| Family [UUDM] | 0.47 | 2.11 | 0.22 | 351 | 0.823 |
| Family [XFDR] | -3.19 | 2.09 | -1.52 | 351 | 0.128 |
| Family [YXFF] | -0.63 | 2.11 | -0.30 | 351 | 0.766 |
| **Random Effects** | | | | | |
| Plant species | 12.00 | | | | |
| Family x Plant species | 1.15 | | | | |
| Inoculation date | 0.00 | | | | |
| *Residual variance* | 21.47 | | | | |

**c.**

|  | **Model 2 (null) - Adult Lifespan** | | | | |
| --- | --- | --- | --- | --- | --- |
| *Fixed effects* | *Estimate* | *SE* | *Statistic* | *df* | *p* |
| (Intercept) | 20.93 | 2.50 | 8.37 | 381 | **<0.001** |
| **Random Effects** | | | | | |
| Plant species | 12.11 | | | | |
| Family x Plant species | 2.64 | | | | |
| Inoculation date | 0.57 | | | | |
| *Residual variance* | 21.51 | | | | |

**d.**

|  | **Model 3 - Adult Lifespan** | | | | |
| --- | --- | --- | --- | --- | --- |
| *Fixed effects* | *Estimate* | *SE* | *Statistic* | *df* | *p* |
| (Intercept) | 23.35 | 0.47 | 49.33 | 380 | **<0.001** |
| Plant species [inc] | -4.95 | 0.54 | -9.20 | 380 | **<0.001** |
| **Random Effects** | | | | | |
| Family | 2.13 | | | | |
| Family x Plant species | 0.87 | | | | |
| Inoculation date | 0.00 | | | | |
| *Residual variance* | 21.55 | | | | |

**e.**

|  | **Model 3 (null) - Adult Lifespan** | | | | |
| --- | --- | --- | --- | --- | --- |
| *Fixed effects* | *Estimate* | *SE* | *Statistic* | *df* | *p* |
| (Intercept) | 20.75 | 0.46 | 44.87 | 381 | **<0.001** |
| **Random Effects** | | | | | |
| Family | 0.00 | | | | |
| Family x Plant species | 9.52 | | | | |
| Inoculation date | 0.00 | | | | |
| *Residual variance* | 21.39 | | | | |

**f.**

|  | **Model 4 - Adult Lifespan** | | | | |
| --- | --- | --- | --- | --- | --- |
| *Fixed effects* | *Estimate* | *SE* | *Statistic* | *df* | *p* |
| (Intercept) | 20.89 | 2.49 | 8.40 | 381 | **<0.001** |
| **Random Effects** | | | | | |
| Plant species | 12.10 | | | | |
| Family | 2.50 | | | | |
| Inoculation date | 0.00 | | | | |
| *Residual variance* | 22.00 | | | | |

**Table S6.** Full model outputs for wing length. a) Model 1, including family, plant species, and their interaction as random effects. b) Model 2, with family as a fixed effect and plant species and the interaction as random effects. c) Model 2₀, the corresponding null model with family removed entirely. d) Model 3, with plant species as a fixed effect and family and the interaction as random effects. e) Model 3₀, the corresponding null model with plant species removed entirely. f) Model 4, identical to Model 1 but with the family by plant species interaction removed.

**a.**

|  | **Model 1 - wing length** | | | | |
| --- | --- | --- | --- | --- | --- |
| *Fixed effects* | *Estimate* | *SE* | *Statistic* | *df* | *p* |
| (Intercept) | 53.35 | 0.14 | 386.04 | 376 | **<0.001** |
| **Random Effects** | | | | | |
| Plant species | 0.00 | | | | |
| Family | 0.37 | | | | |
| Family x Plant species | 0.12 | | | | |
| Inoculation date | 0.00 | | | | |
| *Residual variance* | 1.86 | | | | |

**b.**

|  | **Model 2 - wing length** | | | | |
| --- | --- | --- | --- | --- | --- |
| *Fixed effects* | *Estimate* | *SE* | *Statistic* | *df* | *p* |
| (Intercept) | 53.77 | 0.46 | 117.68 | 347 | **<0.001** |
| Family [BJFO] | -1.77 | 0.74 | -2.41 | 347 | **0.016** |
| Family [BYGA] | 0.25 | 0.64 | 0.39 | 347 | 0.699 |
| Family [CSGQ] | -0.70 | 0.65 | -1.07 | 347 | 0.285 |
| Family [CSLN] | -1.03 | 0.63 | -1.64 | 347 | 0.102 |
| Family [DMHO] | -0.03 | 0.68 | -0.04 | 347 | 0.967 |
| Family [EYJK] | -1.11 | 0.76 | -1.45 | 347 | 0.149 |
| Family [EYNS] | -0.14 | 0.63 | -0.23 | 347 | 0.822 |
| Family [FFJB] | 0.20 | 0.68 | 0.30 | 347 | 0.768 |
| Family [FONX] | 0.22 | 0.64 | 0.34 | 347 | 0.734 |
| Family [FPKE] | 0.23 | 0.67 | 0.35 | 347 | 0.725 |
| Family [GBMN] | -0.14 | 0.70 | -0.20 | 347 | 0.844 |
| Family [HOLR] | -1.85 | 0.62 | -2.98 | 347 | **0.003** |
| Family [HZNP] | -0.71 | 0.68 | -1.05 | 347 | 0.294 |
| Family [HZUU] | 0.23 | 0.74 | 0.31 | 347 | 0.759 |
| Family [JBNS] | -1.00 | 0.65 | -1.54 | 347 | 0.124 |
| Family [KCPM] | -0.66 | 0.69 | -0.95 | 347 | 0.341 |
| Family [KEOV] | -0.73 | 0.66 | -1.12 | 347 | 0.265 |
| Family [KRPQ] | 0.30 | 0.63 | 0.48 | 347 | 0.631 |
| Family [LRSZ] | -0.91 | 0.66 | -1.39 | 347 | 0.167 |
| Family [MNQT] | -0.85 | 0.62 | -1.37 | 347 | 0.173 |
| Family [MOUG] | -0.05 | 0.62 | -0.09 | 347 | 0.931 |
| Family [OVXF] | 0.80 | 0.74 | 1.08 | 347 | 0.279 |
| Family [PQBE] | -1.45 | 0.64 | -2.27 | 347 | **0.024** |
| Family [QTZE] | 0.01 | 0.65 | 0.01 | 347 | 0.992 |
| Family [RLZK] | -0.51 | 0.61 | -0.84 | 347 | 0.404 |
| Family [SZBY] | 0.13 | 0.65 | 0.19 | 347 | 0.846 |
| Family [TTCS] | -2.41 | 0.67 | -3.60 | 347 | **<0.001** |
| Family [UUDM] | 0.13 | 0.65 | 0.20 | 347 | 0.839 |
| Family [XFDR] | 0.98 | 0.65 | 1.50 | 347 | 0.135 |
| Family [YXFF] | -0.49 | 0.65 | -0.77 | 347 | 0.445 |
| **Random Effects** | | | | | |
| Plant species | 0.00 | | | | |
| Family x Plant species | 0.13 | | | | |
| Inoculation date | 0.00 | | | | |
| *Residual variance* | 1.86 | | | | |

**c.**

|  | **Model 2 (null) - wing length** | | | | |
| --- | --- | --- | --- | --- | --- |
| *Fixed effects* | *Estimate* | *SE* | *Statistic* | *df* | *p* |
| (Intercept) | 53.35 | 0.11 | 471.46 | 377 | **<0.001** |
| **Random Effects** | | | | | |
| Plant species | 0.00 | | | | |
| Family x Plant species | 0.47 | | | | |
| Inoculation date | 0.00 | | | | |
| *Residual variance* | 1.87 | | | | |

**d.**

|  | **Model 3- wing length** | | | | |
| --- | --- | --- | --- | --- | --- |
| *Fixed effects* | *Estimate* | *SE* | *Statistic* | *df* | *p* |
| (Intercept) | 53.30 | 0.17 | 322.46 | 376 | **<0.001** |
| Plant species [inc] | 0.09 | 0.17 | 0.52 | 376 | 0.606 |
| **Random Effects** | | | | | |
| Family | 0.36 | | | | |
| Family x Plant species | 0.14 | | | | |
| Inoculation date | 0.00 | | | | |
| *Residual variance* | 1.86 | | | | |

**e.**

|  | **Model 3 (null) - wing length** | | | | |
| --- | --- | --- | --- | --- | --- |
| *Fixed effects* | *Estimate* | *SE* | *Statistic* | *df* | *p* |
| (Intercept) | 53.35 | 0.14 | 386.04 | 377 | **<0.001** |
| **Random Effects** | | | | | |
| Family | 0.37 | | | | |
| Family x Plant species | 0.12 | | | | |
| Inoculation date | 0.00 | | | | |
| *Residual variance* | 1.86 | | | | |

**f.**

|  | **Model 4 - wing length** | | | | |
| --- | --- | --- | --- | --- | --- |
| *Fixed effects* | *Estimate* | *SE* | *Statistic* | *df* | *p* |
| (Intercept) | 53.35 | 0.14 | 387.59 | 377 | **<0.001** |
| **Random Effects** | | | | | |
| Plant species | 0.00 | | | | |
| Family | 0.42 | | | | |
| Inoculation date | 0.00 | | | | |
| *Residual variance* | 1.93 | | | | |
